# Lead and Cadmium Bioaccumulation in Fresh Cow’s Milk in an Intermediate Area of the Central Andes of Peru and Risk to Human Health

**DOI:** 10.3390/toxics10060317

**Published:** 2022-06-11

**Authors:** Doris Chirinos-Peinado, Jorge Castro-Bedriñana, Elva Ríos-Ríos, Gloria Mamani-Gamarra, Elías Quijada-Caro, Analí Huacho-Jurado, Wilfredo Nuñez-Rojas

**Affiliations:** 1 Food and Nutritional Security Research Centre, Universidad Nacional del Centro del Perú, Huancayo 12006, Peru; dchirinos@uncp.edu.pe; 2 Faculty of Science, Universidad Nacional Agraria La Molina, Lima 15024, Peru; erios@lamolina.edu.pe; 3 Ministry of Production, Lima 15036, Peru; gloriagamarra12@gmail.com; 4 Faculty of Zootechnics, Universidad Nacional del Centro del Perú, Huancayo 12006, Peru; elias_256@live.com (E.Q.-C.); anyu_l7@outlook.com (A.H.-J.); 5 Specialized Research Institute, Faculty of Zootechnics, Universidad Nacional del Centro del Perú, Huancayo 12006, Peru; wnunez@uncp.edu.pe

**Keywords:** cadmium, lead, dietary exposure, dietary risk, target hazard quotient, hazard index, contaminated milk

## Abstract

The dairy basin of the Mantaro River located in the centre of Peru faces serious anthropogenic disturbances as it receives emissions and discharges from the metallurgical mining activity located in the headwaters of the basin and milk contaminated with lead (Pb) and cadmium (Cd) endangers the environmental and human health, especially children. To measure the concentrations of Pb and Cd in milk and the dangers of their consumption in the Peruvian population, 40 milk samples were collected and quantified by atomic absorption spectrometry. The mean concentration of Pb in milk was 15 ± 2.6 µg/kg, which represented 75% of the Maximum Limit (ML), and that of Cd was 505 ± 123 µg/kg, which exceeded the ML by more than 194 times. The estimated weekly intake of Pb for people aged 2–85 years was below the Provisional Tolerable Weekly Intake (PTWI) references, determining risk coefficients (CRD) < 1. Weekly Cd intake was much higher than the PTWIs and CRDs were between 14 and 34, indicating that consumers would experience carcinogenic health effects, with children being at higher risk than adults, therefore, milk from the area is not safe for consumption. Cd would be transferred mainly through the soil (water)-grass-milk pathway, due to its presence in irrigation water and in fertilizers that contain Cd. The main pathway for Pb entry would be air-soil (water)-milk grass, from the fine particles emitted into the air by the mining-metallurgical activity, developed approximately 90 km from the study area.

## 1. Introduction

The presence of bioactive peptides, essential amino acids, fat, lactose, calcium, zinc, magnesium, phosphorus, selenium, riboflavin, pantothenic acid, vitamins A, B1, and B12, and other nutrients [1], their therapeutic effects, flavour, and easy digestion make bovine milk widely consumed by the general population.

In children, it benefits their growth, bone development, and health [2,3], is included in the healthy eating guidelines of many countries, and included in school feeding and food assistance programs, and in adults, protective effects on bone health, prevention of chronic diseases, cardiovascular, metabolic, type 2 diabetes and cancer are reported [4,5]. It is an important protein source for populations with limited access to other foods of animal origin.

The beneficial effects of milk can be negated by the bioaccumulation of heavy metals that industrialization and urbanization release into the environment, especially lead (Pb) and cadmium (Cd), toxic metals that easily enter the food chain [6].

Background information on Pb and Cd contents in whole milk in different parts of the world shows large variations associated with the type and level of anthropogenic contamination, the proximity of emission sources, and the type of production system, with the highest contamination problems reported in farms close to mining and metallurgical activities (Table 1 and Table 2).

The average consumption of milk per capita in the world is 100 kg/year, with high variation according to countries/regions. In Peru, by 2020 the per capita milk consumption was 81 kg/year, which is still low in relation to the 130 kg recommended by the FAO [20].

To improve the consumption of milk, its safety and good production practices must be guaranteed throughout the entire production chain, with special care in its contamination by heavy metals such as lead (Pb) and cadmium (Cd), which do not have any biological function and its presence in milk has adverse effects on various organic systems [21,22]. High levels of Pb during pregnancy are associated with lower birth weight and decreased neonatal physiological indicators, which in the long term affect the neuropsychological development of children [9,23,24,25]. Cd intake damages many systems and organs and causes various types of cancer and death [26]. According to the classification of the International Agency for Research on Cancer, a Cd is a Group 1 carcinogen, while inorganic Pb is a group 2 carcinogen [27] and is considered a priority 2 and 7 pollutant in food [28].

Since Pb and Cd have a long exposure life, difficult biodegradation, inadequate decomposition, and high levels of bioaccumulation, their transfer to the food chain makes them very dangerous, and their presence in milk and the risks of their consumption must be evaluated [21,29,30,31,32].

In mining and metallurgical areas located at the headwaters of river basins in the Peruvian Andes, Pb and Cd emissions can exceed the maximum permissible limits (MPL), contaminating atmospheric, water, and soil resources and easily entering the food chain.

Fine particles laden with heavy metals can travel many kilometres through the air and settle in water and soil, bioaccumulate in pastures, and are transferred to crops and other livestock products for human consumption, such as milk [10,33,34].

Additionally, the discharge of mine tailings and industrial and domestic runoff into water sources used for irrigation, the proximity of roads from engine combustion, and the use of phosphorus fertilizers and pesticides containing phosphate rock high in Cd, are sources of milk contamination [33,35,36].

Although there is significant research worldwide on the accumulation of Pb and Cd in milk [6,36], in Peru, there is limited knowledge on the concentration of Pb and Cd in milk and milk products.

In a previous study, we evaluated the risk of Pb and Cd in milk from an area located 20 km from the La Oroya Metallurgical Complex [37] and there are no studies of risks from these metals in areas of greater livestock activity, such as the Mantaro basin, the main source of dairy products for large cities, where the population and the authorities are unaware of the health consequences of the accumulation of Pb and Cd in milk.

Considering that for whole milk Pb should not exceed 20 µg/kg [38,39], and according to the International Dairy Federation, Cd should not exceed 2.6 µg/kg [14,40], the objectives of the study were to determine the concentration of Pb and Cd in milk produced in a livestock area of the Mantaro Valley basin and to assess the risk of the Peruvian population aged 2 to 85 years from exposure to these two metals through milk consumption, providing evidence for the population and management authorities to adopt the necessary measures.

## 2. Materials and Methods

### 2.1. Study Area

In October 2019 and April 2020, 40 milk samples were collected from cows in a livestock area in the central highlands of Peru (Latitude: −11.8219, Longitude: −75.3922; 11°49′19″ S, 75°23′32″ W; 3300 masl), located 92 km from the largest mining-metallurgical industry in the central highlands and one kilometre from the central highway with high interprovincial traffic, an area of intense agricultural activity, whose production is mainly sold in the markets of the Peruvian capital, where more than 30% of the national population is concentrated.

In general, pastures in the area are irrigated with water from the Canal de la Margen Izquierda del Río Mantaro (CIMMIR), which carries water contaminated by different liquid emissions from mining activities and domestic waste. In the study area, the cows are raised in an extensive system with cultivated pastures (*Lolium multiflorum* and *Trifolium repens*), with daily grazing of approximately 9 h.

**Figure 1:**
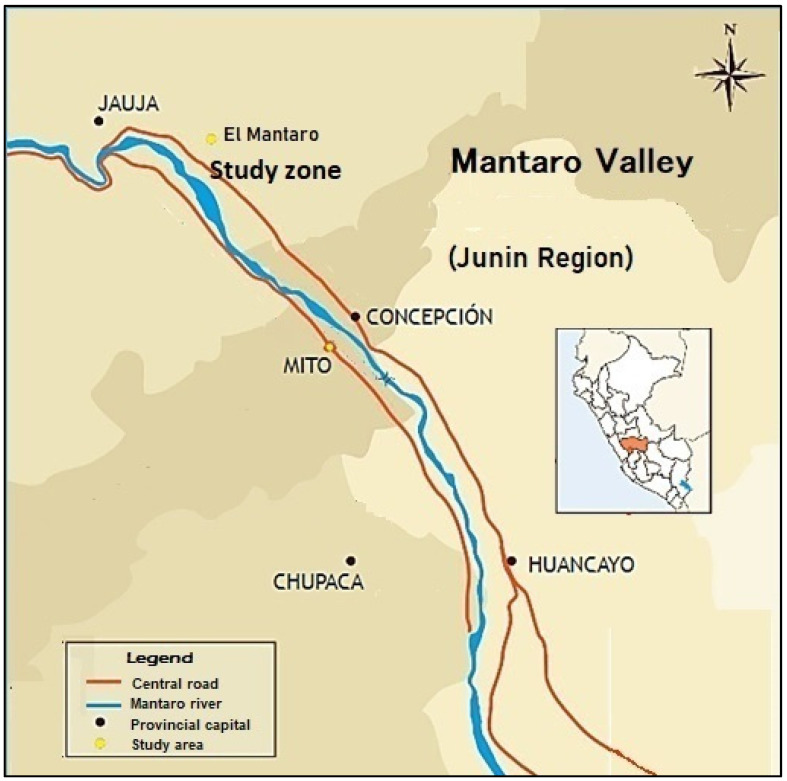
Map of the Mataro Valley—Study Area, Mantaro River left bank.

Figure 1 shows a map showing the location of the study area, an agricultural valley located in central Peru.

### 2.2. Sample Preparation and Analysis

Immediately after milking, milk samples were taken from 20 s and third calving Brown Swiss cows. From each cow, 250 mL of milk were collected following the protocol of the Peruvian Technical Standard 202.112:1998 revised in 2013 [41,42], using sterile polyethene bottles of first use with prior acid wash and rinsed with deionized water, keeping the samples in the cold chain (−18°C) for shipment to the accredited laboratory Baltic Control, Lima-Peru.

Prior to digestion, 50 g of each homogenized sample were placed in porcelain crucibles to be dried at 100 °C to constant weight. They were incinerated in a muffle at 450 °C/15 h, and after cooling they were bleached with 2 mL of 2 N HNO_3_ and dried in thermostatic plates and were re-incinerated at 450 °C/1 h. The ashes were recovered with 20 mL of 0.1 N HNO_3_ and filtered through Watman 40 paper and stored in polypropylene tubes under refrigeration. High purity reagents (Merck KGaA, Darmstadt, Germany) were used.

In the quantification of Pb and Cd, the AOAC 973.35 method was followed, using a flame atomic absorption spectrometer (NAMBEI AA320N), with wavelengths of 283.3 nm and 228.8 nm, respectively, according to the sensitive lines specified in hollow cathode lamps, with Pb and Cd detection limits of 0.0045 and 0.002 mg/kg, respectively [43].

Standards of 1000 mg/kg for each element were used as standards for the calibration curve. To verify the precision of the analytical method, Pb and Cd standard solutions of 155 ± 0.04 and 150 ± 0.05 mg/kg milk were used, and the concentrations of their corresponding runs were 147.14 and 152.50 mg/kg.

The precision of the instrumental methods and analytical procedures was verified by performing duplicate samples. The concentrations of Pb and Cd are expressed in µg/kg.

To provide complementary information, the concentration of Pb and Cd in 6 soil samples and 6 grass samples from the same sampling site was determined using atomic absorption spectrometry. For Pb and Cd, in Peru, the Environmental Quality Standard for agricultural soils refers to a maximum of 70 and 1.4 mg/kg, respectively [44]. Samples were digested using the USEPA method 3050B (SW-846). For digestion, 1 g of the dry sample treated with concentrated nitric acid (HNO_3_) and hydrogen peroxide (H_2_O_2_) was used. Hydrochloric acid (HCl) was added to the initial digest and the sample was heated at reflux to increase metal solubility. The digest was diluted to a final volume of 100 mL.

### 2.3. Risk Assessment

#### 2.3.1. Exposure Assessment (EDI)

Exposure was assessed for the Peruvian population using mean Pb and Cd levels in milk and the mean milk consumption rate published in various studies [45,46,47,48,49]. For the study, the daily milk intake considered for ages 2–5, 6–19, 20–39, 40–59, and 60–85 years of age were 0.500, 0.600, 0.157, 0.154, and 0.195 kg, respectively. The median weights of Peruvian males and females aged 2–85 years correspond to the 2011 Peru Nutritional Status report [50], and there are no more current national reports on this parameter, so it remains valid.

Exposure to Pb and Cd from milk consumption was determined as follows: [15,51,52]:

EDI = CM × DMI/BW,(1)

where EDI is the estimated daily intake of the metal in µg/kg BW/d, BW is the body weight (kg), CM is the metal concentration in milk (µg/kg) and DMI is the daily milk intake (kg).

#### 2.3.2. Dietary Risk Coefficient (DRC)

The daily exposure (μg/day/kg BW) was used to calculate the weekly exposure of each metal (WI: µg/week from milk consumption) for comparison with the provisional tolerable weekly intake (TWI) established for each metal by Joint FAO/WHO Expert Committee on Food Additives (JECFA) [53,54], and the Joint FAO/WHO Expert Committee on Food Additives [55] and World Health, 2012), and to estimate the dietary risk coefficients (DRC) as follows:

DRC = WI/TWI,(2)

where:

DCR is the dietary risk coefficient.

WI is the amount of metal ingested during one week by milk consumption (μg/week).

TWI is the tolerable weekly intake of the metal (μg/week).

A DRC of less than 1 indicates an acceptably low risk, while a ratio greater than 1 indicates a high health risk [56,57].

#### 2.3.3. Target Hazard Quotient (THQ)

The potential chronic non-carcinogenic hazard risk from Pb and Cd from milk consumption was expressed as THQ, and calculated as follows: [58]:

THQ = (EF × ED × DMI × C_M_)/(RfD × ABW × ET), (3)

where

EF is the frequency of exposure to metal per year (365 d).

ED is the exposure period equivalent to the thematic longevity (70 years).

DMI is the daily milk intake (litres)

C_M_ is the concentration of the metal in milk (mg/kg)

RfD is the reference dose for the metal (mg/kg/d)

ABW is the average body weight (60 kg)

ET is the exposure time in days (70 × 365 = 25,550 d)

The reference doses (RfD) for Cd and Pb are 0.001 and 0.0035 mg/kg/d, respectively [50,51,52,53,54,55,56,57,58,59,60,61]. If the THQ is >1, high risk is evident and if <1 there is no risk.

#### 2.3.4. Hazard Index (HI)

The HI was used to assess the potential chronic risk to human health when more than one heavy metal is involved. It represents the long-term risk and was determined by the sum of the hazard quotients (THQ) of the different metals [6,35,62]:

HI = ΣTHQ,(4)

There is no risk to human health if HI < 1 [30,35].

To make comparisons of our findings with other studies, estimates have been made for a person aged 25 years and 60 kg body weight and 16 kg for children [15,63].

#### 2.3.5. Data Processing Techniques

Data were analyzed using Excel-2007 and SPSS (IBM, Endicott, NY, USA) version 23. Pb and Cd contents are expressed as mean ± SD, minimum value, and maximum value. Exposure to these metals was also assessed and weekly intake curves and risk coefficients were generated.

## 3. Results

### 3.1. Concentration of Pb and Cd in the Milk Assessed

The lowest and highest levels of Pb in milk were 10 and 20 µg/kg. For Cd, the values were 280 and 690 µg/kg and had a normal distribution (Table 3).

As complementary information, it is indicated that the average concentrations and standard deviation of Pb and Cd in 6 soil samples from the study area were 49.87 ± 6.27 and 10.13 ± 3.06 mg/kg, and the concentrations of Pb and Cd in the pastures were 5.28 ± 1.89 and 2.74 ± 0.82 mg/kg, respectively.

### 3.2. Dietary Intake and Risk to Pb and Cd from Milk Consumption

Using the average concentrations of Pb and Cd per kilogram of raw milk in the study area (15 and 505 µg) and the average daily milk intake by age in persons aged 2–85 years, the daily and weekly intake of these metals and the corresponding dietary risk were estimated (Table 4).

In children aged 2–5 years, the estimated daily intake of Pb and Cd per kg body weight decreases with age, with younger children being more exposed to the effect of these toxic metals. For the consumption of 500 g of milk per day, the daily intakes of Pb and Cd in children aged 2–5 years were 7.5 and 252 µg, rising until the age of 19 years and then decreasing in adults.

The dietary risk coefficients (DRC) for Pb and Cd in children aged 2 and 5 years were 0.17 and 0.12; while for Cd were 24.6 and 17.0, very high-risk values for this age group (Table 5).

For all ages, the DRCs for Pb are below 1; while for Cd, they are above 1, reaching 24.6 in 2-year-olds, determining that the milk produced in the study area is of very high risk for children and would be unfit for human consumption.

**Figure 2:**
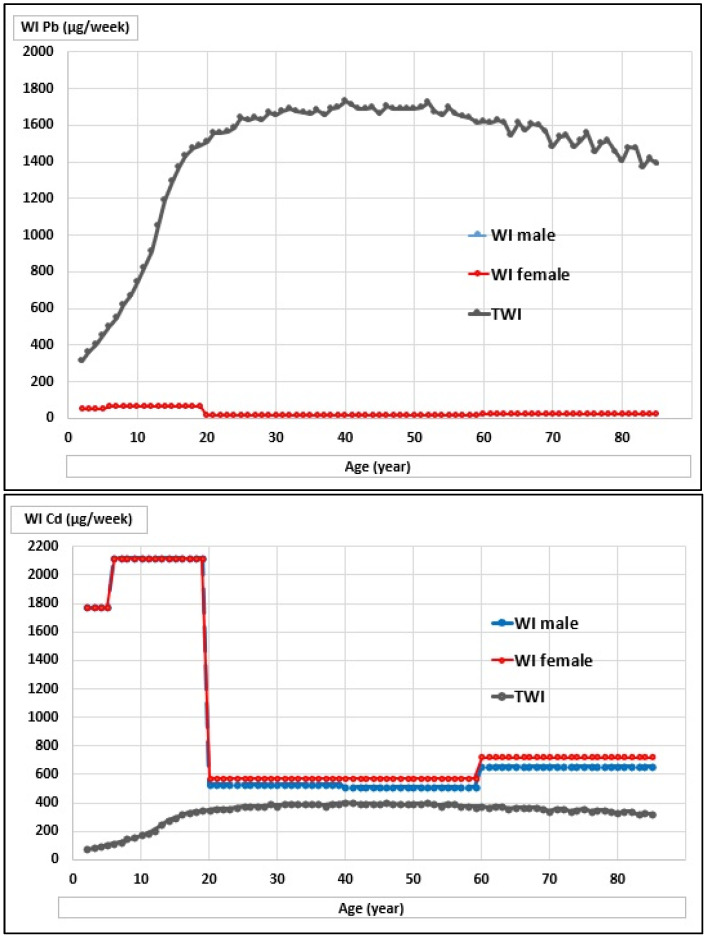
Weekly intake of Pb and Cd by age and sex in relation to the provisional maximum weekly intake (µg). At the top is the Pb WI, and at the bottom is the Cd WI.

**Figure 3:**
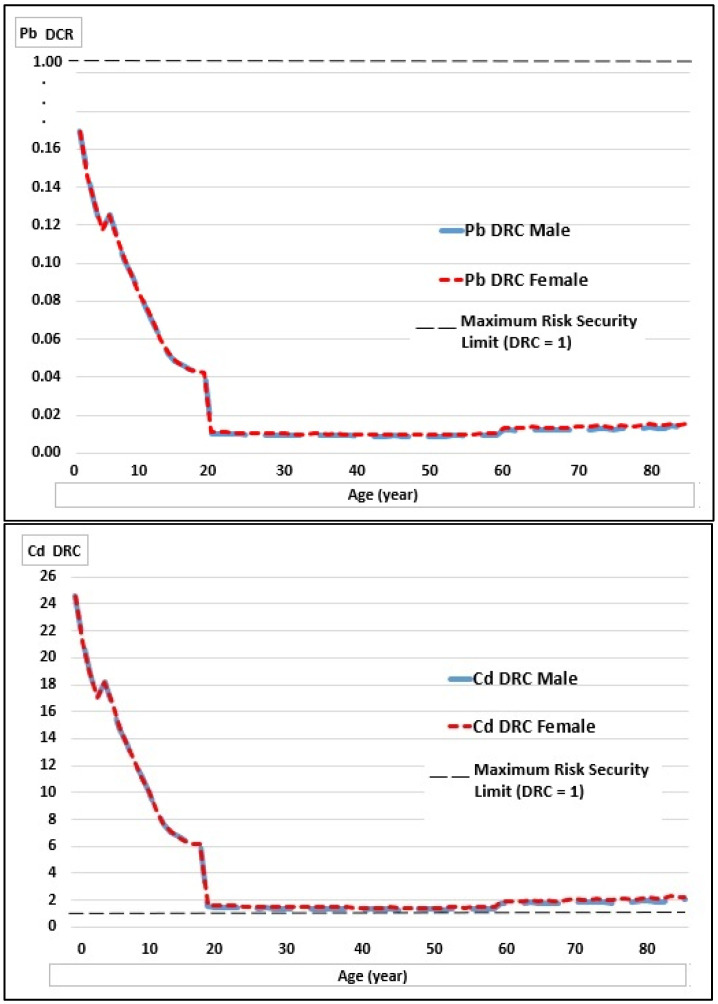
Dietary risk coefficient of Pb and Cd intake by age and sex in relation to the upper safe limit of risk. At the top is the Pb DRC, and at the bottom is the Cd DRC.

Figure 2 and Figure 3 show the weekly intake curves (WI) of Pb and Cd, and the DRC curves by age and sex in relation to the maximum risk safety limit. These curves were constructed with the 2–85-year data to generate continuous lines.

There is a high risk due to the high presence of Cd in the milk produced in the study area.

### 3.3. Target Hazard Quotient (THQ)

To make comparisons of our findings with other studies, we show results determined in 25-year-old adults with a bodyweight of 60 kg [40,53], in this case consuming 150 g of milk per day. The THQs for Pb and Cd were 0.01 and 1.26, respectively.

As the THQ_Cd_ is >1, high risk is evident for 25-year-olds consuming 150 g of milk containing Cd at a concentration of 0.505 mg/kg daily, whereas the THQ_Pb_ < 1 indicates no risk from the intake of this milk.

In children weighing 16 kg, around 4 years of age, who consume 0.5 kg of milk daily, the THQ_Pb_ was 0.134 and the THQ_Cd_ was 15.78, showing a very high risk of Cd for children of this age.

### 3.4. Hazard Index

In this study, the hazard index (HI), which is the sum of the THQ of Pb and Cd per consumption of 150 g of milk for an average 25-year-old person weighing 60 kg was 1.27; of this value, only 0.85% corresponds to Pb and 99.15% to Cd.

In the case of 4-year-old children weighing 16 kg and consuming 500 g of milk/day, the HI is 15.91, with 0.84% corresponding to Pb and 99.16% to Cd, a result that indicates that the main component of the hazard index is given by the presence of Cd in the milk.

## 4. Discussion

### 4.1. Concentration of Pb and Cd in Raw Milk

In this study, Pb and Cd concentrations in whole milk were between 10–20 and 280–690 µg/kg, respectively, values much higher than those reported in studies conducted in other regions of the world, where even milk did not contain Cd [64], and as reported in studies in recent years (Table 1 and Table 2).

In this study the average Pb content has represented 75% of the MPL which is 20 µg/kg [38,39]; however, its prolonged consumption can cause adverse health effects, and behavioural and learning abnormalities in children [65], who absorb 5–10 times more of the ingested Pb than adults [66] and because their developing nervous system is more vulnerable to the heavy metal toxic effects than the adult brain [67].

The average Cd content in the milk samples was 194 times more than the MPL set by International Dairy Federation (2.6 µg/kg) [32,40]. This concentration is well above values reported in Obilić, where the thermal plants “Kosovo A and B” are located, where whole cow’s milk had 40 µg/kg Cd [68].

The high Cd content in milk from the study area could cause serious health problems in the consuming population, damaging different organ systems and giving rise to various types of cancer [26,27,28].

Our results are like those reported in other developing countries where animals consume feed and water contaminated with Pb and Cd from industrial emissions that exceed the maximum limits; while the contents of these metals in milk produced in developed countries, with more controlled industrial activity and more rigorously applied regulations [69], are below those determined in this study.

Higher concentrations of heavy metals in milk are reported in areas close to mining and metallurgical activities, in industrial and high traffic areas, on farms using phosphorus fertilizers, pesticides and fungicides, and when pastures are irrigated with untreated wastewater, canal water loaded with sewage and mining and industrial effluents [70,71].

Lower Pb and Cd concentrations (47 and 4.7 µg/kg) are reported in milk produced in stables near oil fields [15] and industrial areas in Iran (14 and 3.5 µg/kg) [72].

Comparing our results with other studies conducted in the southern highlands of Peru, in the “Coata” river basin, in an area close to emissions and wastewater disposal from mining activity reports Pb and Cd contents of 210 and 3.7 µg/kg [73]. In Umachiri micro-watershed, Puno highlands, the Pb and Cd milk contents exceeded the MPL by 638 and 45 times [74].

In a livestock area located approximately 20 km from the La Oroya Metallurgical Complex, Pb and Cd concentrations of 580 and 20 µg/kg have been reported [10], with Pb contents higher than in the present study, but much lower in Cd. Said area milk has Pb as the main source of contamination present in the dust and vapours emitted by this industry that is deposited in the soil and water.

In our study, the Pb content in the soil (49.87 mg/kg) represented 71% of the maximum level established in the Environmental Quality Standard for agricultural soils [44], and its route of entry into the soil would be through irrigation water from the CIMIRM and fine dust particles and vapours from the mining-metallurgical industry of La Oroya.

In the Cd case, in this study, the soil contained 10.13 mg/kg, 7.2 times higher than the MRL [44], and its pathway of entry into the soil would be mainly due to its presence in phosphorus fertilizers and agrochemicals [75,76,77], in CIMIRM irrigation water and from fine particles that are transported through the air from La Oroya by the action of the winds [78]. This high Cd content in the soil-plant system bioaccumulates in milk, resulting in a high Cd content in this staple food for the Peruvian population.

Phosphorus agrochemicals are based on phosphate rock containing Cd, and triple superphosphate contains 53.2 mg Cd/kg [75,79,80].

### 4.2. Dietary Intake and Risk to Pb and Cd from Milk Consumption

The estimated daily intake of Pb (7.5 µg) in children aged 2–5 years, for daily consumption of 500 g of milk, was 2.5 times higher than the provisional tolerable daily intake estimated by the US Food and Drug Administration-FDA (3 μg/day) and would affect the neurodevelopment of children [81,82,83].

Our result is similar to those reported in farms and markets in Edirne, Turkey, where the intake of toxic metals was below the Turkish Food Codex levels [84], but is much lower than that recorded in children aged 1–7 years in La Plata, Buenos Aires-Argentina, with a daily intake of 138 µg/day and the food groups with the highest Pb intake were processed meat (15.4%), pastries (14.8%), milk (12.5%) and meat (11.7%) [85].

Regarding the weekly reference intake (TWI) of Pb, this increases with age; for children aged 2, 3, 4 and 5 years it is estimated at 300, 375, 455 and 520 µg, and in this study, for the consumption of 500g of milk daily, the values were below the corresponding TWI, determining CRDs below 1, so that this milk from the point of view of its Pb content would not be considered a health risk; however, given the scientific data reporting a number of health problems in children with less than 5 µg/dL of Pb in blood, sustained consumption of this milk may be risky over time [81].

Regarding Cd, in children aged 2–5 years in this study, the estimated daily intake of Cd from consumption of 500 g of milk was 253 µg, a value 44 times higher than the estimated provisional tolerable daily intake of 0.36 μg/kg body weight [54,86], which for children weighing 16 kg would be 5.71 μg.

A provisional tolerable weekly intake (PTWI) of Cd 2.5 μg/kg b.w. guarantees a high level of protection for all consumers, including exposed population subgroups and vulnerable groups [15,54,86]. In our study, for male children aged 2, 3, 4 and 5 years, with average body weights of 12.4, 14.4, 14.4, 16.1 and 17.9 kg, the Cd PTWIs would be 31, 36, 40 and 45 μg/week, respectively, and for 500 g of milk daily consumption these children they would have a weekly intake of Cd of 1764 μg, values 57, 49, 44 and 39 times higher than their corresponding PTWI; therefore, the milk produced in the study region would not be suitable for consumption.

The DRCs for Cd were higher than 1 for all ages, being of higher risk for children because the rates of intake, absorption and accumulation of heavy metals are higher than in adults [24,87,88]; in addition, deficiencies of iron, zinc, and calcium, favour the absorption of Pb and Cd, and deficiencies of B-complex vitamins and vitamin C can exacerbate the adverse effects of Pb poisoning [29].

Regarding THQs, our results in adults weighing 60 kg show a value below 1 for Pb and above 1 for Cd. In children, the THQ was above 15. The HIs were also greater than 1 at all ages, a result of great concern if we consider that simultaneous exposure to two or more metals may have cumulative effects [56,57].

Previous studies carried out in Peru have indicated that milk produced in the vicinity of the mining and metallurgical industry is not suitable for human consumption due mainly to high Pb concentration, while in this study the milk has a high content of Cd. It is important to know the level of contamination of milk by heavy metals and to estimate the dietary risk of its consumption to ensure an adequate intake of safe and innocuous milk. It highlights the importance of the results to be considered in the formulation of standards on maximum levels of these metals in milk and dairy products consumed by the Peruvian population, identifying the main sources of contaminants.

### 4.3. Implications for Peru

In the central area of the Peruvian Andes, there is a traditional dairy farming activity, from family production units to consolidated dairy farms, which for years sold raw milk to large dairy industries; however, in the last decade, the daily collection volume of these companies has been reduced by 73%, generating an available supply of fresh milk for direct consumption or for home industrialization by small informal producers who do not guarantee the safety and quality of the milk and their derivatives.

The bioaccumulation of heavy metals through milk consumption can lead to a variety of toxic effects in a variety of body tissues and organs, especially when Cd is classified by the International Agency for Research on Cancer as carcinogenic to humans (Group 1) [89] and Pb as probably carcinogenic to humans (Group 2 A) [90].

Considering the results of the present study, the 120,000 L per day produced in the Mantaro Valley alone would be unfit for human consumption, due to the potential risk associated with Pb and Cd, especially due to the high content of Cd. [31]. The presence of Pb and Cd in the milk evaluated would have two sources of entry, those coming from emissions to air and water from the mining-metallurgical industry developed in the central highlands of the country, and the metals present in phosphorus fertilizers, mainly Cd, which enter the food chain through the soil-plant-animal system, and which are ultimately transferred to milk and other agricultural products.

Another important aspect of our findings points to the need for review and update of tolerable intake levels of Pb and Cd and strict public measures to minimize contamination of the food chain and reduce the risk to human and animal health from consumption of food contaminated with heavy metals [91].

## 5. Conclusions

This study presents an assessment of the risk for Pb and Cd from the milk consumption produced in a central area of the Peruvian Andes, investigating whether the average consumption of milk in people aged 2 to 85 years constitutes a potentially important source of exposure to these metals and if it poses a health risk.

The average concentration of Pb in the milk samples represented 75% of the maximum limit suggested by the Codex Alimentarius, and the average concentration of Cd was more than 194 times the maximum limit of international standards.

The estimated weekly intake of Pb from the average consumption of milk in the Peruvian population aged 2–85 years does not exceed the references for weekly intake and the dietary risk coefficients were less than 1. The weekly intake of Cd greatly exceeded the references and the risk coefficients ranged between 1.3 and 24.6, being higher in children, which indicates a high potential risk for the consumption of milk produced in the environmental and productive conditions of this region of the world.

Our findings provide scientific evidence for the Peruvian government to establish a monitoring program for the content of Pb and Cd in fresh milk, prior to the establishment of national maximum limits, identifying the sources of contamination and recommending corrective measures to drastically reduce the presence of these metals. heavy in the air, irrigation water, soil, grasses, and other feedstuffs used in Peruvian dairy farming.

## Figures and Tables

**Table 1:** Pb content in whole milk samples from various countries (2016–2022).

Year	Country	Samples (n)	Rank (ug/kg)	Means (ug/kg)	Method	Reference
2022	Bangladeshi	64	0.005–0.02	0.013 ± 0.004	AAS	[7] Hasan et al., 2022
2021	Ecuador	58	1.6–719	208	AAS	[8] De la cueva et al., 2021
2020	Kazajstan	120	1–8	4.5	AAS	[9] Sarsembayeva et al., 2020
2020	Peru	20	540–600	580 ± 18	AAS	[10] Chirinos & Castro, 2020
2020	Russia	1500	75–110	88	AAS	[11] Safonov, 2020
2019	Turkey	20	25–124	-	ICP-MS	[12] Koyuncu & Alwazeer, 2019
2019	Poland		12–13	-	ICP-MS	[13] Sujka et al., 2019
2018	Iran	72	-	32.83 ± 20.8	ICP-OES	[14] Sobhanardakani, 2018
2018	Iran	118	0–250	47 ± 3.9	GFAAS	[15] Norouzirad et al., 2018
2018	Indonesia	30	-	558 ± 43	AAS	[16] Harlia et al., 2018
2016	Iran	50	-	14	Voltametry	[17] Shahbazi et al., 2016
2016	Iran	85	0.7–23.7	3.24 ± 1.32	AAS	[18] Noori et al., 2016

**Table 2:** Cd content in whole milk samples from various countries (2016–2022).

Year	Country	Samples (n)	Rank (ug/kg)	Means (ug/kg)	Method	Reference
2022	Bangladeshi	64	0.021–0.045	0.032 ± 0.005	AAS	[7] Hasan et al., 2022
2020	Kazajstan	120	2.5–2.9	2.7	AAS	[9] Sarsembayeva et al., 2020
2020	Peru	20	11–32	19.7 ± 7.3	AAS	[10] Chirinos & Castro, 2020
2020	Russia	1500	4–11	7.7	AAS	[11] Safonov, 2020
2019	Turkey	20	0.1–4.0	-	ICP-MS	[12] Koyuncu & Alwazeer, 2019
2019	Poland		0–6.7	-	ICP-MS	[13] Sujka et al., 2019
2018	Iran	118	0–100	4.7 ± 1	GFAAS	[15] Norouzirad et al., 2018
2018	Indonesia	30	-	110	AAS	[16] Harlia et al., 2018
2016	Iran	50	-	1	Voltametric	[17] Shahbazi et al., 2016
2016	Bangladeshi	90	<1–75	53	FASS	[19] Muhib et al., 2016

**Table 3:** The concentration of Pb and Cd in milk and comparison with MPLs for whole milk (n = 40).

Variable	Mean	SD	VC, %	Minimum	Maximum	MPL, µg/kg
Pb, µg/kg	15b	2.6	17.90	10	20	20 a
Cd, µg/kg	505 a	123.2	24.41	280	690	2.6 b

a,b, Average values for each metal with different letters vary statistically with its corresponding MPL (*p* < 0.01). MPL: Maximum permissible limit, SD: Standard deviation, CV: coefficient of variation.

**Table 4:** Daily Pb and Cd exposure from milk consumption in populations aged 2–85 years—Perú.

Age (Year)	Body Weight/sex (kg)		DMI (kg)	EDI Pb (µg/kg BW/d)		EDI Cd (µg/kg BW/d)		EDI Pb/d (µg)		EDI Cd/d (µg)	
	Male	Female		Male	Female	Male	Female	Male	Female	Male	Female
2	12.40	11.80	0.500	0.605	0.636	20.36	21.40	7.5	7.5	252	252
5	17.90	17.40	0.500	0.419	0.431	14.11	14.51	7.5	7.5	252	252
10	29.60	29.80	0.600	0.304	0.302	10.24	10.17	9.0	9.0	310	310
15	51.70	49.30	0.600	0.174	0.183	5.86	6.15	9.0	9.0	310	310
20	60.30	53.50	0.157	0.038	0.046	1.27	1.54	2.3	2.4	76	82
30	66.30	59.60	0.157	0.034	0.041	1.15	1.38	2.3	2.4	76	82
40	69.30	61.60	0.154	0.032	0.039	1.06	1.32	2.2	2.4	74	81
50	67.60	60.70	0.154	0.032	0.040	1.09	1.34	2.2	2.4	74	81
60	64.80	59.20	0.195	0.043	0.052	1.44	1.74	2.8	3.1	93	103
70	59.30	54.50	0.195	0.047	0.056	1.58	1.89	2.8	3.1	93	103
80	56.30	52.90	0.195	0.049	0.058	1.66	1.95	2.8	3.1	93	103
85	55.60	49.50	0.195	0.050	0.062	1.68	2.08	2.8	3.1	93	103

DMI: Daily Milk Intake, Kg. EDI: Estimated daily intake.

**Table 5:** Weekly intake and dietary risk to Pb and Cd from milk consumption in populations aged 2–85 years in Peru.

Age (Year)	WI Pb (µg)		WI Cd (µg)		TWI (µg)		DRC Pb		DRC Cd	
	Male	Female	Male	Female	Pb	Cd	Male	Female	Male	Female
2	52.50	52.50	1767	1767	310	72	0.17	0.17	24.6	24.6
5	52.50	52.50	1767	1767	448	104	0.12	0.12	17.0	17.0
10	63.00	63.00	2121	2121	740	172	0.09	0.09	12.4	12.4
15	63.00	63.00	2121	2121	1293	300	0.05	0.05	7.0	7.0
20	15.86	17.12	534	576	1508	350	0.01	0.01	1.5	1.7
30	15.86	17.12	534	576	1658	385	0.01	0.01	1.4	1.5
40	15.86	17.12	534	576	1663	386	0.01	0.01	1.4	1.5
50	15.33	16.90	516	569	1665	386	0.01	0.01	1.3	1.5
60	15.83	16.90	516	569	1695	393	0.01	0.01	1.3	1.5
70	19.43	21.42	654	721	1483	354	0.01	0.01	1.9	2.1
80	19.43	21.42	654	721	1408	327	0.01	0.02	2.0	2.2
85	19.43	21.42	654	721	1390	322	0.01	0.02	2.0	2.2

WI: Weekly intake. TWI: Weekly reference intake. DRC: Dietary Risk Coefficient.

## Data Availability

Data are provided in the article.

## References

[1] Foroutan A., Guo A.C., Vazquez-Fresno R., Lipfert M., Zhang L., Zheng J., Badran H., Budinski Z., Mandal R., Ametaj B.N. (2019). Chemical Composition of Commercial Cow’s Milk. J. Agric. Food Chem..

[2] Marshall T.A., Curtis A.M., Cavanaugh J.E., Warren J.J., Levy S.M. (2018). Higher longitudinal milk intakes are associated with increased height in a birth cohort followed for 17 years. J. Nutr..

[3] Givens D.I. (2020). MILK Symposium review: The importance of milk and dairy foods in the diets of infants, adolescents, pregnant women, adults, and the elderly. J. Dairy Sci..

[4] Alexander D.D., Bylsma L.C., Vargas A.J., Cohen S.S., Doucette A., Mohamed M., Irvin S.R., Miller P.E., Watson H., Fryzek J.P. (2016). Dairy consumption and CVD: A systematic review and meta-analysis. Br. J. Nutr..

[5] Guo J., Astrup A., Lovegrove J.A., Gijsbers L., Givens D.I., Soedamah-Muthu S.S. (2017). Milk and dairy consumption and risk of cardiovascular diseases and all-cause mortality: Dose–response meta-analysis of prospective cohort studies. Eur. J. Epidemiol..

[6] Su C., Gao Y., Qu X., Zhou X., Yang X., Huang S., Han L., Zheng N., Wang J. (2021). The Occurrence, Pathways, and Risk Assessment of Heavy Metals in Raw Milk from Industrial Areas in China. Toxics.

[7] Hasan G.M., Kabir M.H., Miah M.A. (2022). Determination of heavy metals in raw and pasteurized liquid milk of Bangladesh to assess the potential health risks. Food Res..

[8] De la Cueva F., Naranjo A., Puga Torres B., Aragón E. (2021). Presence of heavy metals in raw bovine milk from Machachi, Ecuador (Presencia de metales pesados en leche cruda bovina de Machachi, Ecuador). Rev. Cienc. Vida.

[9] Sarsembayeva N.B., Abdigaliyeva T.B., Utepova Z.A., Biltebay A.N., Zhumagulova S.Z. (2020). Heavy metal levels in milk and fermented milk products produced in the Almaty region, Kazakhstan. Vet. World.

[10] Chirinos-Peinado D., Castro-Bedriñana J. (2020). Lead and cadmium blood levels and transfer to milk in cattle reared in a mining area. Heliyon.

[11] Safonov V. (2020). Assessment of Heavy Metals in Milk Produced by Black-and-White Holstein Cows from Moscow. Curr. Res. Nutr. Food Sci. J..

[12] Koyuncu M., Alwazeer D. (2019). Determination of trace elements, heavy metals, and antimony in polyethylene terephthalate–bottled local raw cow milk of Iğdır region in Turkey. Environ. Monit. Assess..

[13] Sujka M., Pankiewicz U., Kowalski R., Mazurek A., Ślepecka K., Góral M. (2019). Determination of the content of Pb, Cd, Cu, Zn in dairy products from various regions of Poland. Open Chem..

[14] Sobhanardakani S. (2018). Human Health Risk Assessment of Cd, Cu, Pb and Zn through Consumption of Raw and Pasteurized Cow’s Milk. Iran J. Public Health.

[15] Norouzirad R., González-Montaña J.R., Martínez-Pastor F., Hosseini H., Shahrouzian A., Khabazkhoob M., Malayeri F.A., Bandani H., Paknejad M., Foroughi-Nia B. (2018). Lead and cadmium levels in raw bovine milk and dietary risk assessment in areas near petroleum extraction industries. Sci. Total Environ..

[16] Harlia E., Rahmah K.N., Suryanto D. (2018). Food safety of milk and dairy product of dairy cattle from heavy metal contamination. IOP Conf. Ser. Environ. Earth Sci..

[17] Shahbazi Y., Ahmadi F., Fakhari F. (2016). Voltammetric determination of Pb, Cd, Zn, Cu and Se in milk and dairy products collected from Iran: An emphasis on permissible limits and risk assessment of exposure to heavy metals. Food Chem..

[18] Noori N., Noudoost B., Hatami Nia M. (2016). The assessment of lead pollution in milk collected from all dairy farms in Lorestan province, Iran. Toxin Rev..

[19] Muhib M.I., Chowdhury M.A.Z., Easha N.J., Rahman M.M., Shammi M., Fardous Z., Bari M.L., Uddin M.K., Kurasaki M., Alam M.K. (2016). Investigation of heavy metal contents in cow milk samples from area of Dhaka, Bangladesh. Int. J. Food Contam..

[20] León Hinostroza C., MIDAGRI (2021). Anuario Estadístico de la Producción Ganadera y Avícola 2020.

[21] Castro González N.P., Moreno-Rojas R., Calderón Sánchez F., Moreno Ortega A., Meneses M.J. (2017). Assessment risk to children’s health due to consumption of cow’s milk in polluted areas in Puebla and Tlaxcala, Mexico. Food Addit. Contam. Part B Surveill..

[22] López-Rodríguez G., Galván M., González-Unzaga M., Hernández Ávila J., Pérez-Labra M. (2017). Blood toxic metals and hemoglobin levels in Mexican children. Environ. Monit. Assess..

[23] Castro-Bedriñana J., Chirinos-Peinado D., Ríos-Ríos E. (2016). Lead content and placental weight and its association with gestational age, weight, length and hemoglobin in newborns of metallurgical region—Peru. Rev. Toxicol..

[24] Bellinger D.C., Malin A., Wright R.O., Aschner M., Costa L.G. (2018). Chapter One—The Neurodevelopmental Toxicity of Lead: History, Epidemiology, and Public Health Implications. Advances in Neurotoxicology.

[25] Garí M., Grzesiak M., Krekora M., Kaczmarek P., Jankowska A., Król A., Kaleta D., Jerzyńska J., Janasik B., Kuraś R. (2022). Prenatal exposure to neurotoxic metals and micronutrients and neurodevelopmental outcomes in early school age children from Poland. Environ. Res..

[26] Luo H., Gu R., Ouyang H., Wang L., Shi S., Ji Y., Bao B., Liao G., Xu B. (2021). Cadmium exposure induces osteoporosis through cellular senescence, associated with activation of NF-κB pathway and mitochondrial dysfunction. Environ. Pollut..

[27] WHO (2021). IARC Monographs on the Identification of Carcinogenic Hazards to Humans. Agents Classified by the IARC Monographs.

[28] ATSDR (2020). The ATSDR 2019 Substance Priority List.

[29] Ahamed M., Siddiqui M.K. (2007). Environmental lead toxicity and nutritional factors. Clin. Nutr..

[30] Khan K., Khan H., Lu Y., Ihsanullah I., Nawab J., Khan S., Shah N.S., Shamshad I., Maryam A. (2014). Evaluation of toxicological risk of foodstuffs contaminated with heavy metals in Swat, Pakistan. Ecotoxicol. Environ. Saf..

[31] Khan M., Malik R., Muhammad S., Ullah F., Qadir A. (2015). Health risk assessment of consumption of heavy metals in market food crops from Sialkot and Gujranwala districts, Pakistan. Hum. Ecol. Risk Assess. Int. J..

[32] Sobhanardakani S., Tayebi L., Hosseini S.V. (2018). Health risk assessment of arsenic and heavy metals (Cd, Cu, Co, Pb, and Sn) through consumption of caviar of Acipenser persicus from southern Caspian Sea. Environ. Sci. Pollut. Res..

[33] Zhou X., Zheng N., Su C., Wang J., Soyeurt H. (2019). Relationships between Pb, As, Cr, and Cd in individual cows’ milk and milk composition and heavy metal contents in water, silage, and soil. Environ. Pollut..

[34] Castro-Bedriñana J., Chirinos-Peinado D., García-Olarte E., Quispe-Ramos R. (2021). Lead transfer in the soil-root-plant system in a highly contaminated Andean area. PeerJ.

[35] Castro–González N.P., Calderón–Sánchez F., Pérez–Sato M., Soní–Guillermo E., Reyes–Cervantes E. (2019). Health risk due to chronic heavy metal consumption via cow’s milk produced in Puebla, Mexico, in irrigated wastewater areas. Food Addit. Contam. Part B.

[36] Boudebbouz A., Boudalia S., Bousbia A., Habila S., Boussadia M.I., Gueroui Y. (2021). Heavy metals levels in raw cow milk and health risk assessment across the globe: A systematic review. Sci. Total Environ..

[37] Castro-Bedrinana J., Chirinos-Peinado D., Ríos-Ríos E., Machuca-Campuzano M., Gomez-Ventura E. (2021). Dietary risk of milk contaminated with lead and cadmium in areas near mining-metallurgical industries in the Central Andes of Peru. Ecotoxicol. Environ. Saf..

[38] Codex Alimentarius Commission (2011). Report of the 50th Session of the Codex Committee on Food Additives and Contaminants.

[39] European-Union (2015). Commission Regulation (EU) 2015/1005 of 25 June 2015 Amending Regulation (EC) N° 1881/2006 as Regards Maximum Levels of Lead in Certain Foodstuffs. Off. J. Eur. Union.

[40] Malhat F., Hagag M., Saber A., Fayz A.E. (2012). Contamination of Cows milk by heavy metal in Egypt. Bull. Environ. Contam. Toxicol..

[41] NTP (2016). NTP. NTP 202.001. Leche y productos lácteos. Leche cruda. Requisitos. Catálogo de Normas Técnicas Sobre Productos Lácteos.

[42] NTP (2016). 202.112, 1998. Leche y Productos Lácteos. Leche Cruda. Muestreo de Productos Lácteos.

[43] Latimer G.W. (2016). AOAC Official Method 973.35 Lead in Evaporated Milk Atomic Absorption Spectrophotometric Method.

[44] Ministerio del Ambiente (MINAM) 2017. Decreto Supremo N° 011-2017-MINAM. Aprueban Estándares de Calidad Ambiental (ECA) para Suelo (Approval of Environmental Quality Standards (EQS) for Soil). https://sinia.minam.gob.pe/download/file/fid/64487.

[45] Aparco J., Bauista-Olortegui W., Astete-Robilliard L., Pillaca J. (2016). Assessment of nutritional status, dietary intake patterns and physical activity in schoolchildren in the Cercado of Lima. Rev. Peru. Med. Exp. Salud Publica.

[46] USDA (2020). Dairy Update. Country: Peru. United States Department of Agricultura. Foreing Agricultural Service. Report number: PE2020–0024. https://www.fas.usda.gov/data/peru-dairy-update.

[47] Dror D.K., Allen L.H. (2013). Dairy product intake in children and adolescents in developed countries: Trends, nutritional contribution, and a review of association with health outcomes. Nutr. Rev..

[48] Singh G.M., Micha R., Khatibzadeh S., Shi P., Lim S., Andrews K.G., Engell R.E., Ezzati M., Mozaffarian D., Global Burden of Diseases Nutrition and Chronic Diseases Expert Group (NutriCoDE) (2015). Global, regional, and national consumption of sugar-sweetened beverages, fruit juices, and milk: A systematic assessment of beverage intake in 187 countries. PLoS ONE.

[49] Restrepo-Betancur L., Peña-Serna C., Zapata-López N. (2019). Milk Availability of South American Countries in the Last Five Decades: Elements for Analysis and Future Prospects. Inf. Tecnol..

[50] CENAN-INEI (2011). Estado Nutricional en el Perú. Componente Nutricional ENAHO-CENAN-INS.

[51] Christophoridis C., Kosma A., Evgenakis E., Bourliva A., Fytianos K. (2019). Determination of heavy metals and health risk assessment of cheese products consumed in Greece. J. Food Compos. Anal..

[52] Năstăsescu V., Mititelu M., Goumenou M., Docea A.O., Renieri E., Udeanu D.I., Oprea E., Arsene A.L., Dinu-Pîrvu C.E., Ghica M. (2020). Heavy metal and pesticide levels in dairy products: Evaluation of human health risk. Food Chem. Toxicol..

[53] JECFA (2011). Joint FAO/WHO Expert Committee on Food Additives. Evaluation of Certain Food Additives and Contaminants. 73 Report, 2010.

[54] EFSA (European Food Safety Authority) (2012). Cadmium dietary exposure in the European population. EFSA J..

[55] Food and Agriculture Organization, World Health Organization (2012). Safety Evaluation of Certain Food Additives and Contaminants: Prepared by the Seventy Fourth Meeting of the Joint FAO/WHO Expert Committee on Food Additives (JECFA).

[56] Jin Y., Liu P., Sun J., Wang C., Min J., Zhang Y., Wang S., Wu Y. (2014). Dietary exposure and risk assessment to lead of the population of Jiangsu province, China. Food Addit. Contam. Part A.

[57] Juric A.K., Batal M., David W., Sharp D., Schwartz H., Ing A., Fediuk K., Black A., Tikhonov C., Chan H.M. (2018). Risk assessment of dietary lead exposure among First Nations people living on-reserve in Ontario, Canada using a total diet study and a probabilistic approach. J. Hazard. Mater..

[58] Rahmani J., Fakhri Y., Shahsavani A., Bahmani Z., Urbina M.A., Chirumbolo S., Keramati H., Moradi B., Bay A., Bjørklund G. (2018). A systematic review and meta-analysis of metal concentrations in canned tuna fish in Iran and human health risk assessment. Food Chem. Toxicol..

[59] USEPA IRIS (US Environmental Protection Agency’s Integrated Risk Information System) (2011). Environmental Protection Agency Region I, Washington DC. 20460. http://www.epa.gov/iris/.

[60] USEPA (United States Environmental Protection Agency EPA) (2012). Region III Risk-Based Concentration (RBC) Table 2008 Region III, 1650 Arch Street, Philadelphia, Pennsylvania 19103.

[61] USEPA (2021). USEPA Regional Screening Levels (RSLs)—User’s Guide. https://www.epa.gov/risk/regional-screening-levels-rsls-users-guide#toxicity.

[62] Liu X., Song Q., Tang Y., Li W., Xu J., Wu J., Wang F., Brookes P.C. (2013). Human health risk assessment of heavy metals in soil-vegetable system: A multi-medium analysis. Sci. Total Environ..

[63] Islam S., Ahmed K., Al Mamun H., Raknuzzaman M. (2015). The concentration, source and potential human health risk of heavy metals in the commonly consumed foods in Bangladesh. Ecotoxicol. Environ. Saf..

[64] Lante A., Lomolino G., Cagnin M., Spettoli P. (2006). Content and characterisation of minerals in milk and in Crescenza and Squacquerone Italian fresh cheeses by ICP-OES. Food Control.

[65] Akinwunmi F., Akinhanmi T.F., Atobatele Z.A., Adewole O., Odekunle K., Arogundade L.A., Odukoya O.O., Olayiwola O.M., Ademuyiwa O. (2017). Heavy metal burdens of public primary school children related to playground soils and classroom dusts in Ibadan North-West local government area, Nigeria. Environ. Toxicol. Pharmacol..

[66] ATSDR (2019). Lead Toxicity. Case Studies in Environmental Medicine (CSEM). https://www.atsdr.cdc.gov/csem/lead/docs/CSEM-Lead_toxicity_508.pdf.

[67] Neal A.P., Guilarte T.R. (2010). Molecular neurobiology of lead (Pb (2+)): Effects on synaptic function. Mol. Neurobiol..

[68] Andjušić L., Spasić Z., Milošević B. (2012). Influence of industrial air pollutants on the content of cadmium in lucerne and cow milk. Maced. J. Anim. Sci..

[69] Ismail A., Riaz M., Akhtar S., Goodwill J.E., Sun J. (2017). Heavy metals in milk: Global prevalence and health risk assessment. Toxin Rev..

[70] Ismail A., Riaz M., Akhtar S., Ismail T., Amir M., Zafar-ul-Hye M. (2014). Heavy metals in vegetables and respective soils irrigated by canal, municipal waste and tube well waters. Food Addit. Contam. Part B.

[71] Affum A.O., Osae S.D., Kwaansa-Ansah E.E., Miyittah M.K. (2020). Quality assessment and potential health risk of heavy metals in leafy and non-leafy vegetables irrigated with groundwater and municipal-waste-dominated stream in the Western Region, Ghana. Heliyon.

[72] Adbol-Samad A., Nasseri E., Esfarjani F., Mohammadi-Nasrabadi F., Hashemi Moosavi M., Hoseini H. (2020). A systematic review and meta-analysis of lead and cadmium concentrations in cow milk in Iran and human health risk assessment. Environ. Sci. Pollut. Res..

[73] Chata Quenta A. (2015). Presencia de Metales Pesados (Hg, As, Pb y Cd) en el Agua y Leche en la Cuenca del río Coata. Bachelor’s Thesis.

[74] Bárcena L. (2011). Determinación de Metales Tóxicos en la Leche de Ganado Bovino en el Ámbito de la Microcuenca Lechera de Umachiri, Región Puno. Master’s Thesis.

[75] Loganathan P., Hedley M.J., Grace N.D. (2008). Pasture soils contaminated with fertilizer-derived cadmium and fluorine: Livestock effects. Rev. Environ. Contam. Toxicol..

[76] Jiao W., Chen W., Chang A.C., Page A.L. (2012). Environmental risks of trace elements associated with long-term phosphate fertilizers applications: A review. Environ. Pollut..

[77] Oliva M., Camas D.E., Valqui X.J., Meléndez J.B., Leiva S. (2019). Quantitative Determination of Cadmium (Cd) in Soil-Plant System in Potato Cropping (*Solanum tuberosum* var. Huayro). Adv. Agric..

[78] Cullen J.T., Maldonado M.T., Sigel A., Sigel H., Sigel R. (2013). Biogeochemistry of Cadmium and Its Release to the Environment. Cadmium: From Toxicity to Essentiality. Metal Ions in Life Sciences.

[79] Nie X., Duan X., Zhang M., Zhang Z., Liu D., Zhang F., Wu M., Fan X., Yang L., Xia X. (2019). Cadmium accumulation, availability, and rice uptake in soils receiving long-term applications of chemical fertilizers and crop straw return. Environ. Sci. Pollut. Res..

[80] Rigby H., Smith S.R. (2020). The significance of cadmium entering the human food chain via livestock ingestion from the agricultural use of biosolids, with special reference to the UK. Environ. Int..

[81] CDC (2012). Response to Advisory Committee on Childhood Lead Poisoning Prevention Recommendations in “Low Level Lead Exposure Harms Children: A Renewed Call of Primary Prevention”.

[82] Dolan L., Flannery B., Hoffman-Pennesi D., Gavelek A., Jones O., Kanwal R., Wolpert B., Gensheimer K., Dennis S., Fitzpatrick S. (2020). A review of the evidence to support interim reference level for dietary lead exposure in adults. Regul. Toxicol. Pharmacol..

[83] Flannery B., Dolan L., Hoffman-Pennesi D., Gavelek A., Jones O., Kanwal R., Wolpert B., Gensheimer K., Dennis S., Fitzpatrick S. (2019). US Food & Drug Administration’s Interim Reference Levels for Dietary Lead Exposure in Children and Women of Childbearing Age. Regul. Toxicol. Pharmacol..

[84] Bakircioglu D., Topraksever N., Yurtsever S., Kizildere M., Kurtulus Y. (2018). Investigation of macro, micro and toxic element concentrations of milk and fermented milks products by using an inductively coupled plasma optical emission spectrometer, to improve food safety in Turkey. Microchem. J..

[85] Martins E., Malpeli A., Asens D., Telese L., Fasano V., Vargas V., Tavella M., Colman J. (2018). Contribution of diet to lead exposure among children aged 1 to 7 years in La Plata, Buenos Aires. Arch. Argent. Pediatr..

[86] EFSA (2011). European Food Safety Authority, Panel on Contaminants in the Food Chain (CONTAM); scientific opinion on tolerable weekly intake for cadmium. EFSA J..

[87] Lu J., Lan J., Li X., Zhu Z. (2021). Blood lead and cadmium levels are negatively associated with bone mineral density in young female adults. Arch. Public Health.

[88] González N., Calderón J., Rúbies A., Timoner I., Castell V., Domingo J.L., Nadal M. (2019). Dietary intake of arsenic, cadmium, mercury and lead by the population of Catalonia, Spain: Analysis of the temporal trend. Food Chem. Toxicol..

[89] IARC (2017). WHO/IARC (World Health Organization/International Agency for Research on Cancer). List of Classifications, Agents Classified by the IARC Monographs. http://monographs.iarc.fr/ENG/Classification/latest_classif.php.

[90] IARC (Inorganic and Organic Lead Compounds) (2006). IARC Monographs on the Evaluation of Carcinogenic Risks to Humans.

[91] Satarug S., Vesey D., Gobe G. (2017). Health risk assessment of dietary cadmium intake: Do current guidelines indicate how much is safe?. Environ. Health Perspect..

